# Building and evaluating resources for sentiment analysis in the Greek language

**DOI:** 10.1007/s10579-018-9420-4

**Published:** 2018-07-14

**Authors:** Adam Tsakalidis, Symeon Papadopoulos, Rania Voskaki, Kyriaki Ioannidou, Christina Boididou, Alexandra I. Cristea, Maria Liakata, Yiannis Kompatsiaris

**Affiliations:** 1 Department of Computer Science, University of Warwick, Coventry, UK; 2 Information Technologies Institute, CERTH, Thessaloníki, Greece; 3 Centre for the Greek Language, Thessaloníki, Greece; 4 Laboratory of Translation and Natural Language Processing, Aristotle University of Thessaloniki, Thessaloníki, Greece; 5 The Alan Turing Institute, London, UK; 6 Department of Computer Science, University of Durham, Durham, UK

**Keywords:** Sentiment lexicon, Greek language, Word embeddings, Sentiment analysis, Natural language processing, Opinion mining, Emotion analysis, Sarcasm detection

## Abstract

Sentiment lexicons and word embeddings constitute well-established sources of information for sentiment analysis in online social media. Although their effectiveness has been demonstrated in state-of-the-art sentiment analysis and related tasks in the English language, such publicly available resources are much less developed and evaluated for the Greek language. In this paper, we tackle the problems arising when analyzing text in such an under-resourced language. We present and make publicly available a rich set of such resources, ranging from a manually annotated lexicon, to semi-supervised word embedding vectors and annotated datasets for different tasks. Our experiments using different algorithms and parameters on our resources show promising results over standard baselines; on average, we achieve a 24.9% relative improvement in F-score on the cross-domain sentiment analysis task when training the same algorithms with our resources, compared to training them on more traditional feature sources, such as n-grams. Importantly, while our resources were built with the primary focus on the cross-domain sentiment analysis task, they also show promising results in related tasks, such as emotion analysis and sarcasm detection.

## Introduction

During the last decade, the amount of content that is published online has increased tremendously, primarily due to the wide adoption and use of online social media (OSM) platforms. The content produced within OSM has the potential to be used for understanding, modeling and predicting human behavior and its effects. Unsurprisingly, OSM mining has been used in this sense for various tasks, such as trend detection (Aiello et al. 2013), crime rates (Matthew 2014) and election results prediction (Tsakalidis and Papadopoulos 2015), tracking influenza rates (Lampos et al. 2010) and others.

A key task that often needs to be dealt within such problems is *sentiment analysis*—the task of classifying a piece of text with respect to its sentiment, which can be positive, negative or neutral. Other closely related tasks also include *emotion (affect) analysis* and *sarcasm detection* (Gonçalves et al. 2011).

All these tasks are fundamental in order to understand and analyse the public sentiment, emotion or stance around current events and topics of public debate. Despite the fact that a lot of research works on sentiment analysis rely primarily on sentiment lexicons (Ding et al. 2008; Taboada et al. 2011; Navigli and Ponzetto 2012; Mohammad et al. 2013; Zhu et al. 2014), there is not (to the best of our knowledge) any *large-scale* and *systematically evaluated* lexicon for the Greek language.

While there is a great need for generating such a sentiment lexicon for the OSM analysis of Greek text, there are several challenges that arise: works in other languages that create sentiment resources based on SentiWordNet (Esuli andSebastiani 2006) and WordNet synsets (Miller 1995) are not applicable to noisy, user-generated content, such as that of OSM; other works making use of syntactic or part-of-speech (POS) resources (Jijkoun et al. 2010; Vania et al. 2014) cannot be applied on the Greek language, due to the insufficient accuracy of the relevant tools (POS taggers) for Greek. Furthermore, most of the past works evaluate their created resources in a manual fashion, or in a single task (e.g., sentiment analysis); however, real-world multi-task and multi-domain evaluation of sentiment-related resources and comparison with well-established feature baselines are needed in order to demonstrate their effectiveness and generalisation capabilities, as well as their potential weaknesses.

In the current work, we overcome the difficulties stemming from the limited availability of linguistic resources for the Greek language by building upon the definitions of the Greek lemmas of a general lexicon; we present the first publicly available manually annotated Greek Affect and Sentiment lexicon (“GrAFS”); we adapt past methodologies for the English language (Purver and Battersby 2012; Mohammad et al. 2013; Zhu et al. 2014) and, based on our annotations, we create two separate large-scale lexicons for sentiment analysis on social media. We expand our resources based on recent developments in the field of Natural Language Processing, by creating *word embeddings* representations (Goldberg and Levy 2014). We move well beyond the manual evaluation of our resources and provide in-depth analysis of their effectiveness in three different tasks (sentiment and emotion analysis (Mohammad et al. 2017), sarcasm detection) in various datasets using different approaches. Finally, we make all of our resources publicly available for the research community.^1^

## Background

Sentiment analysis in micro-blogging platforms, such as Twitter, is mainly tackled with machine learning techniques, rather than by the use of lexicons (Gonçalves et al. 2013). Yet, lexicon-based methods have proven sufficient when dealing with sentiment analysis, as they can achieve an important level of coverage (Gonçalves et al. 2013) and can render very high precision rates (Khan et al. 2015). Moreover, they seem to be more effective when applied across domains and can better handle negation and intensification (Taboada et al. 2011), as well as improve the performance of opinion retrieval systems (Jijkoun et al. 2010).

Past works on generating lexical resources in non-English languages has primarily relied on translations of English-based sentiment lexicons and mappings of WordNet synsets, to transfer the polarised words from English to the target language (Jijkoun et al. 2010; Das and Bandyopadhyay 2010; Arora et al. 2012; Perez-Rosas et al. 2012); while common tools for expansion methods of the generated lexicon include part-of-speech (POS) taggers (Vania et al. 2014) and syntactic rules (Jijkoun et al. 2010). In particular, Das and Bandyopadhyay (2010) used the Subjectivity Word List (Wilson et al. 2005) and leveraged WordNet synsets to create a lexicon for the Indian languages, which was further expanded using a corpus-based approach. In Vania et al. (2014), a similar approach was used for generating an initial lexicon for the Indonesian language, which was expanded using different methods, such as finding words in common patterns of three-grams with positive/negative words in a corpus. Perez-Rosas et al. (2012) showed that bridging the language gap between English and Spanish languages using the multilingual sense-level aligned WordNet structure allows to generate a high accuracy polarity lexicon. Other approaches include a PageRank-like algorithm that was used in Jijkoun and Hofmann (2009) for creating a lexicon in Dutch based on the relations of the WordNet synsets; synonym and antonym relations have been used for expanding a lexicon for Hindi by Arora et al. (2012), while the use of word affixes has also been exploited by Mohammad et al. (2009). With respect to generating resources specifically for the Greek language, Palogiannidi et al. (2015) translated English words from the ANEW lexicon (Bradley et al. 1999) and manually annotated them with respect to their valence, arousal and dominance. Other works on sentiment-related tasks in the Greek language have not created and comparatively evaluated linguistic resources for such tasks (Agathangelou et al. 2014; Solakidis et al. 2014).

As there do not exist any reliable syntactic parsing and POS tagging tools for the Greek language, making use of such resources (Jijkoun et al. 2010; Vania et al. 2014) is not possible in our case, while language-dependent word-level rules (Mohammad et al. 2009) cannot generalise; also, translation techniques and WordNet synset mapping (Jijkoun et al. 2010; Das and Bandyopadhyay 2010; Arora et al. 2012; Perez-Rosas et al. 2012) are risky and ineffective when dealing with noisy content. Furthermore, none of the above works has evaluated the generalisation capabilities of the generated resources with respect to different tasks from different domains. Other approaches, such as translating the documents from the target language into English, have shown surprising improvements in performance of sentiment analysis models (Mohammad et al. 2016), but those are expensive and cannot be applied with high confidence in a highly inflected language, such as Greek. Last but not least, to the best of our knowledge, the only work that has focused on the Greek language, by Palogiannidi et al. (2015), created a lexicon of words with respect to their valence, arousal and dominance and not to their sentiment or emotional orientation. While such emotional dimensions of a word might indeed be helpful in a sentiment classification task, they are not as explicit as the standard subjectivity and polarity labels of the words for the sentiment analysis task.

## Generating the resources

Here we present the three lexicons that have been created. We first present the manually annotated lexicon (“GrAFS”) that was generated using the online version of Triantafyllides’ Lexicon (1998), as a starting point (Sect. 3.1). Then, we present the automatically generated sentiment lexicons (Sect. 3.2) and the word embeddings representations (Sect. 3.3).

### GrAFS lexicon creation

The lexicon by Triantafillidis (1998) is one of the largest and widely recognised general dictionaries existing for the Modern Greek language, counting 46,747 lemmas. One of its distinctive features is that, despite the fact that it has been designed for human use, it seems to have been conceived to promote NLP tasks, as it standardises linguistic data (e.g., nouns are organised in declension classes, descriptions are given in a systematic way, without comments or assumptions). Furthermore, in its electronic version, as provided by the Centre for the Greek Language,^2^ all information types are tagged (e.g., part of speech, declension class, example, etymology, use, register of language, semantic field), making it the largest existing lexical resource of that type for use in NLP tasks in the Greek language. In order to aggregate words that could possibly contain sentimental load, we crawled the electronic version of the lexicon. In particular, we used the advanced search utilities to retrieve all words that can be used in an ironic (346 words), derogatory (458), abusive (90), mocking (31) or vulgar tone (53). Furthermore, since the electronic version of this lexicon provides the capability to search through the description of every word, we further searched these descriptions for emotional words (e.g., feel).^3^

The above process resulted in the collection of 2324 words and their definitions. Those were then manually annotated with respect to their expressed sentiment and affect. The annotators were four of the authors of the paper—two with a Computer Science and two with a Linguistics background. Every annotator was first asked to annotate each word as objective, or strongly or weakly subjective. If subjective, then the annotator would assign a polarity label to the word (positive/negative/both) and rate it with respect to its affect on an integer scale from 1 (does not contain this affect at all) to 5 along Ekman’s six basic emotions (anger, disgust, fear, happiness, sadness, surprise) (Ekman 1992). In all annotations (subjectivity, polarity and the six emotions), the annotators were allowed not to rate a word at all if they were not sure about its meaning and use. We also created extra columns for comments and proposed synonyms for every word, but did not use those fields for the purpose of this work. These annotations have been previously released; however, no systematic evaluation has been performed on them up to now.

**Table 1:** Annotators’ agreement for subjectivity (Pearson correlation), positive and negative (Cohen’s Kappa), respectively

(a) Subjectivity				(b) Positive				(c) Negative			
	#2	#3	#4		#2	#3	#4		#2	#3	#4
#1	.47	.90	.77	#1	.40	.82	.51	#1	.28	.85	.45
#2		.45	.59	#2		.38	.45	#2		.31	.42
#3			.60	#3			.53	#3			.47

**Table 2:** Annotators’ agreement (Pearson correlation) for the six emotions

(a) Anger				(b) Disgust				(c) Fear			
	#2	#3	#4		#2	#3	#4		#2	#3	#4
#1	.28	.68	.55	#1	.47	.74	.57	#1	.37	.60	.35
#2		.34	.39	#2		.45	.53	#2		.41	.28
#3			.58	#3			.56	#3			.46

(d) Happy				(e) Sad				(f) Surprise			
	#2	#3	#4		#2	#3	#4		#2	#3	#4
#1	.42	.83	.62	#1	.40	.59	.47	#1	.18	.50	.17
#2		.40	.53	#2		.39	.46	#2		.18	.40
#3			.62	#3			.53	#3			.20

Then, we eliminated words for which there was a missing subjectivity score for more than one annotator, reducing our lexicon to 2260 words. We corrected the few entries that were judged as objective but had a non-zero polarity or emotional score, by converting the positive and negative scores to 0 and the emotion scores to 1 (that is, their minimum allowed score), since these entries were judged to be wrongly annotated, as they were not in line with the annotation instructions. We also converted the subjectivity scores to three values: 0 for objective, .5 for weakly subjective and 1 for strongly subjective. Finally, we averaged the subjective, positive, negative and the six emotion scores as provided by the annotators. The annotators’ agreement is shown in Tables 1 and 2. We measure the agreement in terms of Cohen’s Kappa for the positive and negative dimensions, since these form two distinct classes; for the rest, we measure the agreement in terms of Pearson correlation. We notice a fair agreement (.40–.60) in most cases, with the exception of the surprise dimension. The reason behind this is probably the nature of the surprise emotion, which, in contrast to the rest, can be expressed both in a positive and negative way, thus challenging the annotators.

**Fig. 1:**
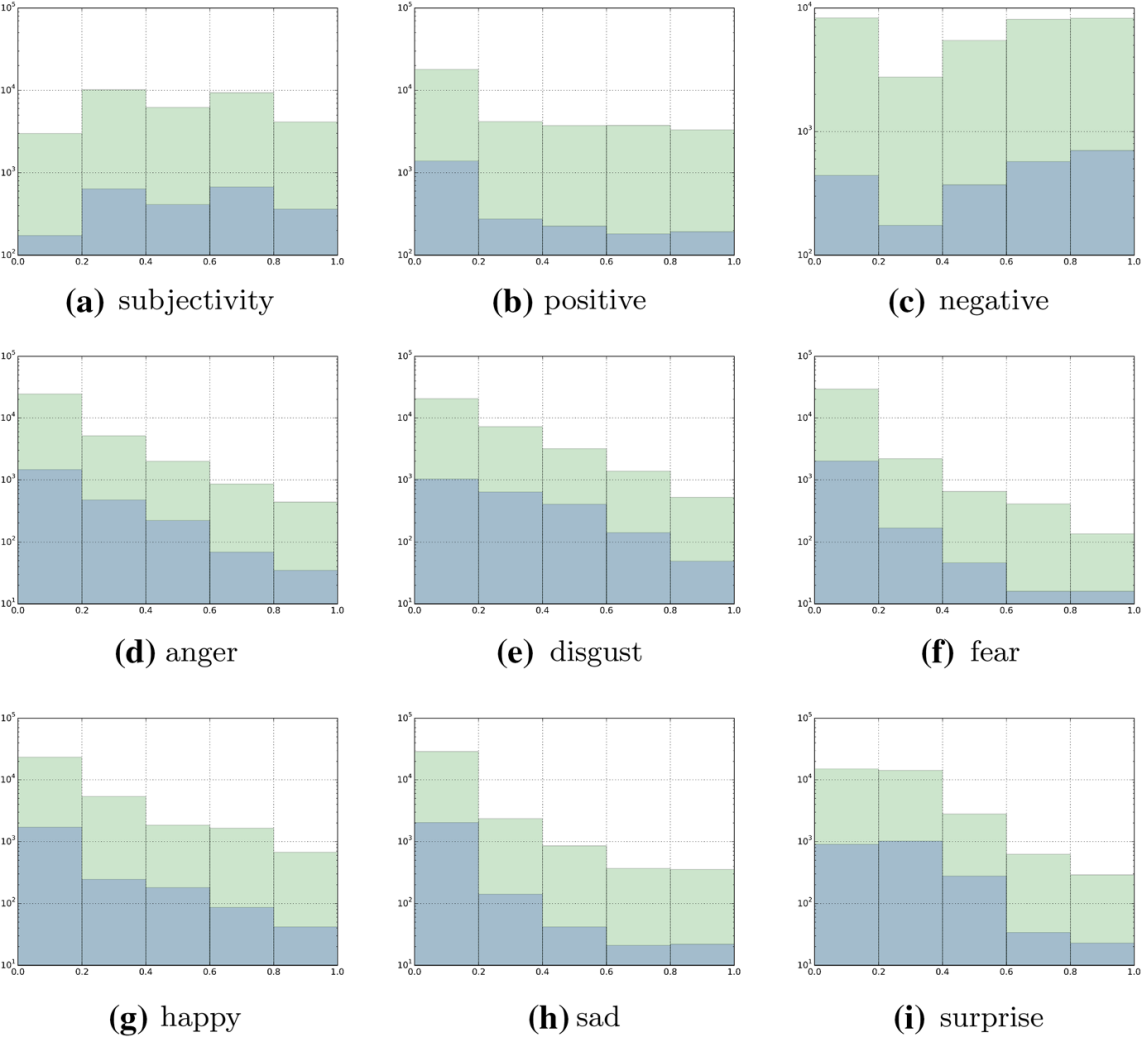
Distributions (in log scale) of word scores before (blue) and after (green) the morphological expansion. **a** Subjectivity, **b** positive, **c** negative, **d** anger, **e** disgust, **f** fear, **g** happy, **h** sad and **i** surprise. (Color figure online)

Since the Greek language is a highly inflected language, the next step was to produce all inflected forms derived from the extracted lemmas. This task was performed semi-automatically, using NLP tools developed by the Laboratory of Translation and Natural Language Processing for Greek language analysis (Constant and Yannacopoulou 2003; Kyriacopoulou 2004), thus expanding the list of our keywords using all declension and conjugation classes derived from the original words and replicating their sentiment and emotion scores. The final version of the lexicon after this process consists of 32,884 unique inflected forms.^4^ Figure 1 displays the distributions of the scores before and after the morphological expansion (for the six emotions, we normalised the scores in the [0, 1] range). What is noticeable is that the distributions are not affected by the expansion: the lower Pearson correlation between them is observed for the case of “Negative” sentiment (.89); for the rest of sentiments and emotions, the respective correlation is > .95. Furthermore, it is shown that there are more negative than positive words, while the majority of the words do not carry a strong emotional value, as indicated by the annotators.

### Twitter-specific sentiment lexicons

A common drawback of applying a sentiment lexicon in user-generated content is that, due to the informal nature of the content, it is difficult to find exact matches of the keywords in the lexicon. For that reason, we created two Twitter-specific lexicons that have the potential to capture a larger portion of sentiment-related keywords as expressed in social media, including misspellings, abbreviations and slang.

Given a set of positive ($D_{{ pos}}$) and negative ($D_{{ neg}}$) documents composing a corpus *D* with $D_{{ pos}}\cup D_{{ neg}} = D$ and $D_{{ pos}}\cap D_{{ neg}} = \emptyset$, a common practice to find the degree of association of each n-gram *n* appearing in *D* with each sentiment class (pos, neg) is to calculate the pointwise mutual information (PMI) of *n* with respect to each class and use Eq. (1) to assign a score ${ sen}$ to it (Mohammad et al. 2013):

1 $$\begin{aligned} {\text {sen}}(n) = {\text {PMI}}(n,{ pos}) - {\text {PMI}}(n,{ neg}), \end{aligned}$$

where ${\text {PMI}}(n, {cls}) = \log (p({ cls}|n)/p({ cls}))$ for each class *cls* = {*pos*, *neg*}. This process results in a dictionary that associates each n-gram with a sentiment score. Then, feature extraction from a document can take place based, for example, on the summation of the n-grams’ sentiment scores. While the lexicons that have been created for the English language using this methodology have proven to be quite effective (Mohammad et al. 2013; Zhu et al. 2014), the task of creating a large-scale annotated Greek corpus to serve as *D* is quite difficult and time consuming. To deal with this issue, we used two semi-supervised methods and created two Twitter-specific lexicons. For both, we used the Twitter Streaming API,^5^ in order to collect tweets in the Greek language. Then, we followed some common preprocessing steps [tokenisation (Gimpel et al. 2011), lowercasing, replacement of user mentions with usrmention and of URLs with urlink, removal of non-alphanumeric characters and of one-character-long unigrams] and calculated the score of every n-gram appearing at least 10 times in *D*, according to Eq. (1).

#### Keyword-based lexicon (KBL)

We collected about 15 million tweets in Greek (excluding retweets) over a period of more than 2 months (August–November 2015) constrained on the occurrence of at least one of 283 common Greek stop words.^6^ In order to create our corpus *D*, positive and negative words from GrAFS were used as seeds. This stems from our assumption that a tweet containing a polarised keyword would lead to the respective sentiment for the whole tweet. We consider a positive (negative) word as a positive (negative) seed word if (*a*) its subjectivity score in the GrAFS lexicon is at least 0.75, (*b*) its positive (negative) score is 1.0 and (*c*) its negative (positive) score is 0. In this way, we extracted words with clearly positive and negative sentiment (based on our annotations), ending up with 1807 positive and 4852 negative seed words. Intuitively, relaxing the previous constraints would yield more, yet noisier, seed words; for that reason, we avoided using such an approach. Using our seed words, and not taking into consideration the short tweets in our collected data (${ length}<25$ characters), we found 593,321 positive and 340,943 negative tweets in our corpus. We excluded tweets appearing in both positive and negative tweet sets, resulting in a dataset of 892,940 tweets to be used as the corpus for generating our first Twitter-based lexicon. After the preprocessing steps mentioned above, we were left with 190,667 n-grams (52,577 unigrams, 138,090 bigrams) comprising our Keyword-based lexicon (KBL).

#### Emoticon-based lexicon (EBL)

A practice that is commonly followed in sentiment analysis in OSM in order to create large-scale training sets is to search for tweets containing emoticons and assign them the corresponding sentiment or emotional label (Go et al. 2009; Purver and Battersby 2012; Tsakalidis et al. 2014). We followed this procedure, collecting tweets containing emoticons of the six basic emotions (Ekman 1992) as in Purver and Battersby (2012), over a period of 5 months (January–June 2015). Only tweets containing happy- and sad-related emoticons were in reasonable quantity to serve our purposes (about 200K/25K tweets with happy/sad emoticons, respectively), under the restrictions of being non-retweeted tweets and of a minimum length of 25 characters. Following the exact same procedure as with the KBL lexicon, we created the new lexicon (EBL) containing 32,980 n-grams (14,424 unigrams, 18,556 bigrams).

The method for creating the two Twitter-based lexicons is the same (only the corpus changes). Indeed, we found that 88% of the n-grams that are included in EBL, are also present in KBL. Interestingly, the Pearson correlation between the co-occuring terms is only 29.5%. The reason for this is that the corpus of creating the EBL lexicon is noisier and smaller compared to the KBL. In an attempt to quantify the noise contained in our lexicons, we compiled a list of 634 stop words^7^ and found that many of them are included in our lexicons with some sentiment score (485 in KBL; 414 in EBL). Other cases, such as negation, are also not explicitly handled by our lexicons. For example, 1.9% of the entries in KBL (2.7% in EBL) are n-grams that contain one of the five most popular negation words in Greek $({\upmu \upeta (\upnu ), \updelta \upvarepsilon (\upnu ), \acute{\hbox {o}} \upchi \upiota })$, with the majority of them (62% in KBL; 70% in EBL) having negative scores. We consider dealing with such linguistic cases as part of our future work.

### Twitter-specific word embeddings

While sentiment lexicons have shown a great potential when applied on OSM data, they still do not capture the context of a keyword: a sentiment score is assigned to every n-gram, regardless of the context it is being used. Most importantly, n-grams are represented as different discrete symbols, providing us with no information of the similarity of their meaning. To address this limitation, dense word representations have been proposed to capture the context in which they appear and have gained ground over the latest years (Turian et al. 2010). Recent advances have made it possible to tackle this problem by representing every word as a vector of values (“word embedding”), which is generated through various methods, such as neural networks or dimensionality reduction on the word co-occurrence matrix (Mikolov and Dean 2013; Mikolov et al. 2013; Goldberg and Levy 2014).

To assess the effectiveness of such representations in the Greek language, we applied word2vec using the skip-gram architecture (Mikolov and Dean 2013) in our corpus of 15M tweets that was used for creating KBL.^8^ The selection of word2vec was based on its wide and successful application in many NLP tasks, while the selection of the skip-gram architecture was based on its ability to deal with rare dictionary words that appear quite often in social media due to their noisy nature. We followed the same pre-processing steps as with our lexicons, set the minimum frequency of unigrams to 5 and used a 5-token window around every word. We opted for a smaller number of word occurrences compared to the lexicons (5 vs. 10) since word2vec produced context-aware word representations, thus requiring smaller number of training examples compared to the co-occurrence-based method of generating our lexicons. Then, we created word embeddings of length $n=300$ ($|V|=418{,}402$). Further increasing the length of the vector representations would have led to a high increase in computational cost during the learning process, while there is not sufficient evidence in literature that a larger length would also imply an increase in accuracy for sentiment-related tasks.

An alternative way of generating such latent representations would have been to train a neural network on a labeled (positive/negative) corpus (Kalchbrenner et al. 2014)—e.g., by using the corpus used for EBL with positive/negative emoticons. However, this would have been based on a much smaller corpus, resulting in task-specific representations that might not be as effective in other tasks. We have also tried to build representations derived from word2vec using the sentiment-specific corpora from which our lexicons were built; however, we noticed that the accuracy dropped in the experiments that follow in the next sections, compared to the one obtained by using the full-corpus word2vec representations. The reason for this is that the sizes of the corpora that were used for creating the KBL/EBL lexicons were much smaller than the 15M tweets corpus (890K/225K, respectively), thus providing word2vec with much less contextual information about the words, leading into qualitatively poorer word embeddings representations.

## Experimental setup

To evaluate our resources, we performed several experiments, using different algorithms on three different sentiment-related tasks, as follows:

- *Task 1 (sentiment analysis)* Given a tweet, classify it as positive, negative or neutral (classification task).
- *Task 2 [Emotion (intensity) analysis (Mohammad et al.* 2017*)]* Given a tweet, find the level for each of the conveyed emotions, on a 0–5 scale (regression task).
- *Task 3 (Sarcasm detection)* Given a tweet, classify it as being sarcastic or not (binary classification task).

### Datasets

#### Task 1

We worked on three different datasets for the sentiment analysis task, as presented in Table 3. The first two (“TIFF”, “TDF”) were acquired from Schinas et al. (2013) and consist of tweets in Greek and English, concerning the Thessaloniki Film Festival and Thessaloniki Documentary Festival respectively. In our experiments, we focused strictly on the tweets written in Greek.^9^ The third dataset (“GRGE”) consists of tweets related to the January 2015 General Elections in Greece, extracted by providing the streaming API with a keyword list of the main political party names, their abbreviations and some common misspellings. All duplicates were excluded and 2309 tweets (randomly selected) were annotated with respect to their sentiment. Each tweet was annotated by two MSc graduates (one with Engineering and one with Economics background) and native Greek speakers, who were selected based on their keen interest in the elections in order to ensure good annotation quality. The annotators were asked to detect the sentiment of the author of the tweet. In rare cases of presence of both positive and negative sentiment within the same tweet, the annotators were instructed to annotate it based on the prevailing sentiment. The Cohen’s kappa coefficient over the initial set of 2309 tweets was 0.525. Hence, we only kept the ones (1640) for which there was an agreement.

**Table 3:** Number of tweets per-class in the sentiment analysis task

	Positive	Neutral	Negative	Total
TIFF	876	1566	314	2756
TDF	786	813	228	1827
GRGE	79	979	582	1640

#### Task 2

For the emotion analysis task we used the dataset made available by Kalamatianos et al. (2015). It consists of 681 tweets annotated by two annotators with respect to their emotion on a scale from 0 to 5. Due to the low agreement between the annotators for the angry and disgust emotions, we excluded them from our analysis; for the rest, we consider the average emotion score given by the two annotators as our ground truth.

#### Task 3

To the best of our knowledge, there does not exist a publicly available dataset for sarcasm detection in the Greek language. Therefore, we created a new annotated dataset, consisting of tweets related to the Greek General Elections of January, 2015. A random set of 3000 tweets were annotated with respect to being sarcastic or not. Every tweet was annotated by the same annotators as the GRGE dataset (sarcastic/non-sarcastic—or N/A, if the annotator was uncertain); we then removed all the tweets that were marked as N/A and only kept the ones for which there was an agreement (2506 overall, Cohen’s kappa coefficient: 0.76). Note that, as expected, the majority of tweets (79.3%) belong to the non-sarcastic class (1988 vs. 518).

### Feature extraction

We used three different sets of features which are extensively used in sentiment-related tasks in the English language. Before performing feature extraction, we applied the same pre-processing steps as for the lexicon generation (lowercasing, replacing URLs and usernames, tokenising and removing all non-alphanumeric characters). Note that some of these steps might actually hurt accuracy in sentiment-related tasks (e.g., an all-uppercase word in a tweet might be indicative of the tweet sentiment); we leave the assessment of such features as part of our future research. We did not perform stop word removal or stemming, since those steps were found to have no or negative influence on the sentiment analysis tasks (Bermingham and Smeaton 2010; Saif et al. 2012) and we had to be consistent with the way that our lexicons were previously created. The feature sets that were extracted are the following:

#### Ngrams (N)

For each of our tasks, we extracted unigrams and bigrams with binary values, excluding n-grams that appeared only once in the training set.

#### Lexicons (L)

We mapped every unigram and bigram to both KBL and EBL and extracted the following features: the number of positive (negative) matches of every unigram and bigram in the lexicons (that is, the total count of unigrams/bigrams with associated lexicon score larger—for positive—and smaller—for negative—than zero), the total sum (float) of positive (negative) unigrams and bigrams scores and the overall summation of their respective scores. We also extracted the same features regardless of whether they referred to unigrams or bigrams. This led to a total number of 30 features per tweet. Finally, using the initial GrAFS lexicon, we extracted the overall sum of the unigrams’ subjective, positive and negative scores, as well as the six emotions, leading to a total number of 39 features.

#### Word embeddings (E)

We mapped every word of every tweet to its word embeddings vector. In order to represent every tweet in these vector spaces, we applied three functions on every dimension of its words’ vectors (${ min}, { max}$ and ${ mean}$) (Tang et al. 2014), leading to 900 features for every tweet. Other functions, such as the summation or the multiplication, could have also been used; however, finding the optimal type of functions to use was considered out of the scope of this work.

Each of these feature sets was examined separately in our experiments. We also created representations, by merging each pair (**“NL”**, **“NE”**, **“EL”**), as well as all of them together (**“NLE”**). These seven representations were provided separately as input to our classifiers in the three tasks, to examine their effectiveness when used alone and in conjunction with each other. To get further insights on the quality of our resources, we also compare the performance for the same tasks and with the same setup when using features derived strictly from *(a)* our GrAFS lexicon (“${\bf L}_g$”), *(b)* the Twitter-specific lexicons (“${\bf L}_{tw}$”) and *(c)* an automatically translated sentiment lexicon for the English language (“${\bf L}_{tr}$”). For the latter, we employed the popular Emotion Lexicon by Mohammad and Turney (2010) and Saif (2013), which contains annotations of English words with respect to 10 affect dimensions (subjective, positive, negative, angry, anticipation, disgust, fear, happy, sad, trust), 7189 of which have been automatically translated into Greek using Google Translate.^10^ The features are extracted by summing the number of unigram/bigram occurrences for each dimension of every tweet.

### Classification and regression algorithms

To explore the use of our resources in depth, we employed three algorithms for the classification tasks (Tasks 1 and 3). These were the logistic regression (LR), random forests (RF) and support vector machines (SVM) with an RBF kernel. Every algorithm was tested on each set of features for all tasks using 10-fold cross validation. In order to study the cross-domain effectiveness of our features on Task 1, we also performed experiments by training on the feature sets of every two datasets and testing on the third. For the regression task (Task 2), we opted to use the least absolute shrinkage and selection operator (LASSO), random forests for regression (RFR) and support vector regression (SVR). Due to the small size of the dataset in Task 2, we opted for a 5-fold cross-validation (to avoid having folds of very small size).

We did not perform parameter optimisation in any of the tasks, as finding the optimal parameters or algorithms was out of the scope of the current work; however, we did run our experiments with different parameters (the $\alpha$ parameter for LASSO, the number of trees for RF/RFR and the *C* parameter in SVM/SVR). For LASSO, we performed our experiments with different values for the $\alpha$ parameter ranging from $10^{-5}$ to $10^{3}$; for SVM and SVR we performed experiments with *C* varying from $10^{-5}$ to $10^{3}$; for RF and RFR, we performed our experiments with 100 up to 1000 trees, with increases of 100. Only the results of the algorithms with the best-performing parameters are reported; however, there were not major deviations in the results of any algorithm under different parameters observed in any task (except for extreme cases of *C* in SVM/SVR).

We have also compared the results obtained by the classification algorithms (Tasks 1, 3) against the majority class baseline (MC). For the regression task (Task 2), we defined our baselines as *(a)* the average ground-truth predictor MC$_{{ avg}}$ and *(b)* the model MC$_{{ dist}}$ that predicts an emotion score for an instance randomly, yet based on the probability distribution of the ground-truth; for the latter, we performed 1000 experiments and report here average statistics for every emotion.

## Results

### Task 1: Sentiment analysis

We used the weighted-average F-measure for the evaluation of Task 1. This was selected due to its nature of being a harmonic mean between precision and recall, while weighted-averaging was preferred over macro-averaging, in order to avoid a biased estimation of the algorithms’ performance, due to the limited amount of positive examples in the GRGE dataset. Results are presented per dataset and per algorithm, as well as macro-averaged (across the three datasets). We are also presenting the majority classifier (MC) as our baseline.

Table 4 presents the results obtained using 10-fold cross validation on the three datasets. The comparison between our two lexicons shows that our expanded L$_{tw}$ lexicon captures domain-specific sentiment features better than L$_{g}$, probably due to its larger size, whereas better performance is achieved consistently on average when these two resources are merged (L). Importantly, all of our lexicon resources outperform the translated L$_{{ tr}}$ lexicon by a clear margin. From the six individual representations, n-grams (N) and word embeddings (E) consistently outperform all the lexicon-based representations. Despite that, our lexicons can be used effectively alongside with both representations, yielding a slightly better performance than the individual L/E models. However, the main advantage of the lexicon (L) and word embeddings (E) representations is their cross-domain nature, which is studied next.

**Table 4:** F-measure based on 10-fold cross-validation for Task 1

Dataset	Model	Baselines		Our resources				Combinations			
		N	L$_{tr}$	L$_g$	L$_{tw}$	L	E	NL	NE	LE	NLE
TIFF	MC	41.15	41.15	41.15	41.15	41.15	41.15	41.15	41.15	41.15	41.15
	LR	61.35	42.75	55.32	56.29	57.83	59.56	**63.29**	60.28	62.28	62.49
	RF	56.93	44.20	57.99	56.08	59.54	59.79	59.90	59.00	**61.51**	60.62
	SVM	59.52	43.99	58.00	48.31	49.73	61.96	62.11	62.53	63.58	**64.34**
TDF	MC	27.36	27.36	27.36	27.36	27.36	27.36	27.36	27.36	27.36	27.36
	LR	62.64	42.48	51.22	53.87	54.17	60.56	**65.87**	62.27	61.86	63.23
	RF	58.85	45.96	52.05	54.67	59.18	62.40	62.45	62.42	**63.97**	63.85
	SVM	60.24	46.05	51.64	53.65	53.75	63.29	63.75	63.22	65.28	**66.53**
GRGE	MC	44.63	44.63	44.63	44.63	44.63	44.63	44.63	44.63	44.63	44.63
	LR	80.37	52.11	60.86	72.52	72.46	76.72	**80.66**	77.82	77.55	78.06
	RF	**79.35**	53.35	65.32	71.43	73.19	78.14	76.42	78.01	78.28	77.98
	SVM	79.17	52.82	62.76	68.30	68.44	**80.65**	79.36	79.71	80.32	79.72
avg	MC	37.71	37.71	37.71	37.71	37.71	37.71	37.71	37.71	37.71	37.71
	LR	68.12	45.78	55.80	60.89	61.49	65.61	**69.94**	66.79	67.23	67.93
	RF	65.04	47.84	58.45	60.73	63.97	66.78	66.26	66.48	**67.92**	67.48
	SVM	66.31	47.62	54.47	56.75	57.31	68.63	68.41	68.49	69.73	**70.20**

The best performing feature set per algorithm is highlighted in bold

The domain-dependence of the n-grams representation (N) is clearly illustrated in Table 5. For comparison purposes, we have also included the relative decrease obtained in the cross-domain experiments when compared to the corresponding intra-domain ones that were presented in Table 4. The performance of our algorithms when trained on n-grams from the other two datasets drops by 28.29% on average, compared to the 10-fold cross-validation approach. This highlights the importance of using features that can be used in a cross-domain fashion, so that one does not need manually annotated data for all possible domains, in order to develop an accurate sentiment classifier. L$_{{ tr}}$ can barely outperform the majority classifier (MC); on the contrary, our manually annotated L$_{g}$ lexicon is the most robust representation. Word embeddings form again the best-performing individual feature set, followed by our lexicon-based features. Those two combined (LE) yield the best across-algorithm and across-datasets results; the incorporation of n-grams on top of them has a slightly negative effect on the performance on average (except for the case of SVM). This is an important finding for the cross-domain sentiment analysis task also, because it indicates that the use of a relatively small, fixed number of features can yield better results, alleviating the learning models from the task of dealing with the sparse bag-of-words representations that have a negative effect on the accuracy, while increasing the computational cost. Finally, it should be noted that the accuracy of the best performing feature set in the GRGE dataset drops much more than the accuracy on TDF and TIFF, if we compare those against the results obtained by 10-fold cross-validation (from 80.66 to 63.71). The reason behind this effect is that the TDF/TIFF datasets are related (documentary and film festivals respectively), as opposed to the GRGE. Thus, the performance achieved in GRGE represents a more realistic evaluation of our resources in a completely new domain.

**Table 5:** F-measure based on cross-domain experiments for Task 1

Test set	Model	Baselines		Our resources				Combinations			
		N	L$_{tr}$	L$_g$	L$_{tw}$	L	E	NL	NE	LE	NLE
TIFF	MC	41.15	41.15	41.15	41.15	41.15	41.15	41.15	41.15	41.15	41.15
	LR	53.56	42.58	57.88	57.54	58.43	58.90	59.93	58.26	**60.20**	58.46
	RF	54.55	44.74	56.68	55.32	57.20	62.64	60.08	61.35	**63.73**	63.00
	SVM	51.42	44.20	57.14	47.49	49.47	60.45	61.56	61.09	61.30	**63.32**
TDF	MC	27.36	27.36	27.36	27.36	27.36	27.36	27.36	27.36	27.36	27.36
	LR	44.01	28.81	44.45	50.41	51.96	56.11	**59.81**	54.14	57.28	56.17
	RF	34.20	31.37	47.40	50.40	53.02	50.86	49.16	43.85	**54.76**	46.34
	SVM	40.68	31.30	47.38	36.57	38.06	59.03	56.42	59.51	59.51	**61.02**
GRGE	MC	44.63	44.63	44.63	44.63	44.63	44.63	44.63	44.63	44.63	44.63
	LR	51.14	45.79	49.20	56.63	56.49	60.06	55.90	56.43	**61.32**	59.22
	RF	46.17	46.62	49.85	58.03	**58.97**	48.27	52.84	48.46	51.27	48.13
	SVM	53.56	46.38	51.61	45.68	47.31	**63.71**	62.01	63.19	57.07	63.04
avg	MC	37.71	37.71	37.71	37.71	37.71	37.71	37.71	37.71	37.71	37.71
	LR	49.57	39.06	50.51	54.86	55.63	58.36	58.55	56.28	**59.60**	57.95
	RF	44.97	40.91	51.31	54.58	56.40	53.92	54.03	51.22	**56.59**	52.49
	SVM	48.55	40.63	52.04	43.25	44.95	61.06	60.00	61.26	59.29	**62.46**
relative decrease (%)	LR	27.23	14.68	**9.48**	9.90	9.53	11.05	16.29	15.74	11.35	14.69
	RF	30.86	14.49	12.22	**10.13**	11.83	19.26	18.46	22.95	16.68	22.21
	SVM	26.78	14.68	**4.46**	23.79	21.57	11.03	12.29	10.56	14.97	11.03

The first column indicates the test dataset, after training the models on the rest

The best performing feature set per algorithm is highlighted in bold

### Task 2: Emotion intensity analysis

We used the mean squared error (MSE) and Pearson’s correlation coefficient ($\rho$) as the evaluation measures for this task. These are popular for the evaluation of regression tasks, measuring the error by putting more weight on the larger errors (MSE) and the correlation between the predicted and the actual scores, respectively.

Tables 6 and 7 show the results using 5-fold cross-validation. “Fear” is the emotion for which all models achieve the lowest error rates, albeit barely outperforming our baseline model MC$_{{ avg}}$; Pearson correlation is also low, due to the low variance of values in the dataset for this emotion. For the rest of the emotions, the results reveal a similar difficulty level with each other in terms of predicting their values. In all cases, our features clearly outperform the N and L$_{{ tr}}$ baselines.

For clearer comparison, Table 8 presents the cross-emotion results (MSE, $\rho$); in particular, we present the macro-average evaluation metrics across all algorithms and emotions, as well as the macro-average metrics, by selecting the best algorithms per emotion and representation (e.g., SVR’s $\rho =.388$ is selected against LASSO and RFR for the “happy” emotion for the N representation). Intuitively, the selection of the best algorithm for every emotion is crucial in a real-world application, thus the comparison of the best algorithms per representation in Table 8 is of great importance.

The comparison between the different features reveals that the lexicon features L$_{{ tw}}$ and L clearly achieve the lowest error rates on average; however, it is the word embeddings and the combined representations using them that outperform the rest with respect to $\rho$. Note that the MC$_{{ avg}}$ has an MSE-average of 1.72, which is equal to the MSE-best of L$_{{ tr}}$, demonstrating the inability of the latter to capture the emotion contained within a tweet. The comparison between our lexicons shows that L$_{g}$ performs poorly compared to L$_{{ tw}}$ (probably due to the noisy language of social media, which is better captured by L$_{{ tr}}$), whereas their combination into L does not boost performance for this task. Overall, the comparison of the best models per emotion and per representation reveals that our word embeddings form the best representation for this task and a small boost in accuracy is provided when our lexicon features are used alongside them (LE). This is an important finding, as it shows that our resources can provide a relative improvement of 13.5% in MSE rates (28.4% in $\rho$) over the most competitive pre-existing baseline (N), despite the fact that they were built with a primary focus on the task of sentiment analysis.

**Table 6:** MSE for the emotion prediction task (Task 2), using 5-fold cross validation

Emotion	Algorithm	Baselines		Our resources				Combinations			
		N	L$_{tr}$	L$_{g}$	L$_{tw}$	L	E	NL	NE	LE	NLE
Fear	MC$_{avg}$	0.68	0.68	0.68	0.68	0.68	0.68	0.68	0.68	0.68	0.68
	MC$_{dist}$	1.35	1.35	1.35	1.35	1.35	1.35	1.35	1.35	1.35	1.35
	LASSO	0.88	0.70	0.69	**0.67**	0.68	0.98	0.85	0.77	0.98	0.78
	RFR	0.73	0.73	0.73	0.67	0.68	0.71	**0.66**	0.67	0.70	0.67
	SVR	0.69	0.73	0.75	0.69	0.71	0.67	0.73	0.73	**0.66**	0.71
	Average	0.77	0.72	0.72	**0.68**	0.69	0.79	0.75	0.72	0.78	0.72
Happy	MC$_{avg}$	2.08	2.08	2.08	2.08	2.08	2.08	2.08	2.08	2.08	2.08
	MC$_{dist}$	4.17	4.17	4.17	4.17	4.17	4.17	4.17	4.17	4.17	4.17
	LASSO	2.42	2.09	1.93	1.92	**1.87**	2.61	2.48	2.28	2.60	2.26
	RFR	1.94	2.06	1.87	1.72	1.69	1.57	1.68	1.57	**1.56**	1.57
	SVR	1.87	2.20	2.05	1.65	1.69	**1.62**	1.93	1.78	1.62	1.72
	Average	2.08	2.12	1.95	1.76	**1.75**	1.93	2.03	1.88	1.93	1.85
Sad	MC$_{avg}$	1.98	1.98	1.98	1.98	1.98	1.98	1.98	1.98	1.98	1.98
	MC$_{dist}$	3.98	3.98	3.98	3.98	3.98	3.98	3.98	3.98	3.98	3.98
	LASSO	2.35	2.00	1.92	**1.89**	1.91	2.80	2.28	2.11	2.80	2.07
	RFR	1.82	2.07	1.95	1.77	1.71	1.58	1.68	1.58	1.58	**1.58**
	SVR	1.85	2.75	2.87	1.81	1.87	**1.65**	2.09	1.81	1.66	1.80
	Average	2.01	2.27	2.25	**1.82**	1.83	2.01	2.02	1.83	2.01	**1.82**
Surprise	MC	2.12	2.12	2.12	2.12	2.12	2.12	2.12	2.12	2.12	2.12
	MC$_{dist}$	4.19	4.19	4.19	4.19	4.19	4.19	4.19	4.19	4.19	4.19
	LASSO	2.82	2.13	2.12	**1.96**	1.99	3.22	2.75	2.3	3.16	2.28
	RFR	1.82	2.18	2.10	1.72	1.67	1.57	1.63	1.56	1.57	**1.56**
	SVR	1.87	2.36	2.24	1.88	1.95	1.79	2.02	1.87	**1.68**	1.82
	Average	2.17	2.22	2.15	**1.85**	1.87	2.19	2.13	1.91	2.14	1.89

The best performing feature set per algorithm is highlighted in bold

**Table 7:** Pearson correlation for the emotion prediction task (Task 2), using 5-fold cross validation

Emotion	Algorithm	Baselines		Our resources				Combinations			
		N	L$_{tr}$	L$_{g}$	L$_{tw}$	L	E	NL	NE	LE	NLE
Fear	MC$_{avg}$	.000	.000	.000	.000	.000	.000	.000	.000	.000	.000
	MC$_{dist}$	.000	.000	.000	.000	.000	.000	.000	.000	.000	.000
	LASSO	.200	-.020	.043	.119	.092	.148	.213	**.243**	.162	.226
	RFR	.192	.007	.086	.214	.203	.188	**.266**	.222	.192	.225
	SVR	.197	.022	.146	.210	.196	.276	.135	.239	**.278**	.240
	Average	.196	.003	.092	.181	.164	.204	.205	**.235**	.211	.230
Happy	MC$_{avg}$	.000	.000	.000	.000	.000	.000	.000	.000	.000	.000
	MC$_{dist}$	.000	.000	.000	.000	.000	.000	.000	.000	.000	.000
	LASSO	.345	.099	.276	.283	.324	.353	.341	.360	.353	**.364**
	RFR	.370	.162	.343	.429	.446	.499	.458	.498	**.502**	.501
	SVR	.388	.158	.287	.471	.462	**.501**	.409	.468	.495	.463
	Average	.368	.140	.302	.394	.411	**.451**	.403	.442	.450	.443
Sad	MC$_{avg}$	.000	.000	.000	.000	.000	.000	.000	.000	.000	.000
	MC$_{dist}$	.000	.000	.000	.000	.000	.000	.000	.000	.000	.000
	LASSO	.311	.071	.184	.218	.213	.267	.322	.355	.263	**.361**
	RFR	.357	.061	.226	.346	.376	.452	.400	.453	.453	**.453**
	SVR	.358	.094	.161	.346	.327	**.443**	.249	.409	.428	.395
	Average	.342	.075	.190	.303	.305	.387	.324	**.406**	.381	.403
Surprise	MC$_{avg}$	.000	.000	.000	.000	.000	.000	.000	.000	.000	.000
	MC$_{dist}$	.000	.000	.000	.000	.000	.000	.000	.000	.000	.000
	LASSO	.265	.067	.084	.277	.258	.259	.272	.376	.269	**.385**
	RFR	.417	.073	.226	.442	.465	.513	.480	.519	.517	**.521**
	SVR	.370	.031	.143	.399	.388	.449	.364	.415	**.482**	.451
	Average	.351	.057	.151	.373	.370	.407	.372	.437	.423	**.452**

The best performing feature set per algorithm is highlighted in bold

**Table 8:** Cross-emotion results for Task 2

Emotion	Baselines		our resources				Combinations			
	N	L$_{tr}$	L$_{g}$	L$_{tw}$	L	E	NL	NE	LE	NLE
MSE-average	1.76	1.83	1.77	**1.53**	1.54	1.73	1.73	1.59	1.72	1.57
MSE-best	1.55	1.72	1.65	1.45	1.44	1.35	1.41	1.35	**1.34**	1.35
$\rho$-average	.314	.069	.184	.313	.313	.362	.326	.380	.366	**.382**
$\rho$-best	.341	.088	.235	.368	.377	.436	.401	.428	**.438**	.429

The best performing feature set per algorithm is highlighted in bold

### Task 3: Sarcasm detection

Table 9 presents the F-score on a per-class and a macro-average basis. We include the per-class results, in order to study them in more detail, with an emphasis on the sarcastic class.

Overall, there are small differences observed in the F-score for the non-sarcastic class, apart from the individual L$_{{ tr}}$, L$_{g}$ lexicon-based representations, which perform the worst for almost all algorithms. The latter is also the case for the sarcastic class, in which the lexicon-based representations perform very poorly. On the one hand, this might imply that our lexicons are unable to deal with sarcasm. On the other hand, given that sarcasm detection is a rather context-dependent task, this might also mean that our lexicons’ contribution to this task should be evaluated in a cross-domain manner, similar to Task 1. Nevertheless, both L$_{g}$ and L$_{tw}$ confidently outperform L$_{tr}$, whereas merging them into L yields consistently better results than the individual L$_{g}$ and L$_{tw}$ for all algorithms and classes. Word embeddings, on the other hand, outperform all lexicon-based approaches in almost all cases and form a competitive feature source against n-grams for this task.

The comparison between the rest of the resources shows that there is a small improvement when combining different feature sets over n-grams or word embeddings. Overall, the best macro-average score is achieved by SVM, when trained on word embeddings and n-gram features, outperforming the best n-gram-based model by almost 1%. While this improvement is relatively small, it is worth noting that those results are achieved using 10-fold cross-validation on the same dataset and not in a different domain, in which the n-grams tend to perform a lot worse in sentiment-related tasks, as demonstrated in Table 5. Cross-domain sarcasm detection is a challenging direction for future work.

**Table 9:** F-score on the Sarcasm detection task

		Baselines		Our resources				Combinations			
Class	Model	N	L$_{tr}$	L$_{g}$	L$_{tw}$	L	E	NL	NE	LE	NLE
Non-sarcastic	MC	88.47	88.47	88.47	88.47	88.47	88.47	88.47	88.47	88.47	88.47
	LR	92.75	88.48	88.76	91.00	91.21	90.87	**92.79**	91.97	91.33	91.85
	RF	92.93	88.51	88.73	90.11	90.42	93.01	91.59	92.65	**92.96**	92.81
	SVM	92.34	88.49	88.59	87.20	87.22	92.64	92.30	**93.46**	92.28	93.40
Sarcastic	MC	0.00	0.00	0.00	0.00	0.00	0.00	0.00	0.00	0.00	0.00
	LR	70.94	0.77	22.43	57.70	59.05	64.52	**71.37**	67.93	66.21	67.92
	RF	**71.61**	12.11	33.43	50.72	52.10	68.50	59.72	65.53	68.84	67.04
	SVM	72.32	11.79	21.70	33.99	39.31	68.63	71.50	**73.14**	68.50	73.10
Macro-average	MC	44.23	44.23	44.23	44.23	44.23	44.23	44.23	44.23	44.23	44.23
	LR	81.85	44.63	55.59	74.35	75.13	77.69	**82.08**	79.95	78.77	79.88
	RF	**82.27**	50.31	61.08	70.41	71.26	80.76	75.65	79.09	80.90	79.93
	SVM	82.33	50.14	55.14	60.60	63.26	80.64	81.90	**83.30**	80.39	83.25

The best performing feature set per algorithm is highlighted in bold

### Key findings

Our results demonstrate the effectiveness of our resources in all studied tasks. While the accuracy that is expected using our resources in a particular task may vary (i.e., due to the limited resources in the Greek language, we were restricted to five datasets overall), the boost in performance when employing our lexicons and embeddings are consistent in all cases. Overall, our main findings with respect to the effectiveness of our resources in the three studied tasks are summarized as follows:

1. In the intra-domain sentiment analysis and sarcasm detection tasks, the n-gram representation is hard to beat. This is expected, since n-grams form a competitive representation due to their nature of capturing word-to-class associations within a single domain, under the assumption that such information (i.e., domain-specific annotations) are available. Nevertheless, by using strictly our resources or our resources alongside the n-gram feature set for the sentiment analysis task, we obtain an average (across-datasets) relative improvement of 2.7–5.6%, depending on the algorithm used. For sarcasm detection, the differences in F-score for our resources in comparison with the n-gram baseline are minor, primarily due to the context-dependent nature of the task, which is captured effectively by the n-grams.
2. On the contrary to the above finding, in the emotion detection task, the n-gram representation is performing quite poorly, achieving the lowest correlation and highest error rates when compared to our lexicons and word embeddings. We achieve 9.5% improvement in Pearson correlation and 0.2 error reduction rates, by using only our word embedding representation, whereas the addition of other features yields only minor differences in terms of accuracy. The reason for this effect is that the emotion intensity task was not studied on a single domain; hence, our word embeddings, which are trained over a large and generic corpus, form a more appropriate feature extraction method for this type of task.
3. The major advantage of our resources is highlighted in the cross-domain sentiment analysis task, which is the task that motivates the creation of such resources. Given that it is impossible to have annotated datasets for all domains and purposes, creating lexicons and resources that can be used in a new domain is of crucial importance in sentiment analysis. Here we demonstrated that we achieve a clear improvement in accuracy (24.9% relative improvement on average, across the three algorithms in Table 5) over the best n-gram model. Importantly, a similar improvement (22.7% across the three algorithms) results from using features derived strictly from our resources, again improving the computational load of any algorithm.
4. Finally, in all tasks, we observe that our GrAFS lexicon consistently outperforms the translated one. However, our Twitter-based lexicons (KBL, EBL) form much better feature extraction resources for all tasks, clearly demonstrating the importance of building resources for handling user-generated content, which is not captured by our expanded GrAFS lexicon. Nevertheless, we plan to investigate whether the same conclusion holds when dealing with more well-formed documents, such as news articles.

## Conclusion

In this paper we presented the generation and evaluation of various rich resources for sentiment-related analysis for the Greek language. We have evaluated our resources in-depth with very promising results. Importantly, our evaluations moved beyond the popular sentiment analysis task, demonstrating the effectiveness of our resources in multiple related tasks, including *emotion* and *sarcasm detection*. We plan to use our resources for the real-time monitoring of the Greek Twittersphere and expand our evaluation to the task of stance detection. By releasing our resources, we aspire to encourage and support research on sentiment-related tasks in the Greek language.

## Footnotes

1 The resources are available at: mklab.iti.gr/resources/tsakalidis2017building.zip.

2 http://www.greek-language.gr/greekLang/modern_greek/tools/lexica/index.html.

3 The exact words that were used and the number of words found are: $\upsigma \upupsilon \upnu \upalpha \acute{\upiota } \upsigma \upvartheta \upeta \upmu \upalpha \, (603), \upalpha \upiota \upsigma \upvartheta \acute{\upalpha } \upnu \hbox {o} \upmu \upalpha \upiota \, (154), \upalpha \acute{\upiota } \upsigma \upvartheta \upeta \upsigma \upeta \, (121), \upalpha \acute{\upiota } \upsigma \upvartheta \upeta \upmu \upalpha \,(793), \upsigma \upupsilon \upnu \upalpha \acute{\upiota } \upsigma \upvartheta \upeta \upsigma \upeta \,(17), \upalpha \upiota \upsigma \upvartheta \acute{\upalpha } \upnu \upvarepsilon \uptau \upalpha \upiota \, (88), \upnu \upiota \acute{\upomega } \upvartheta \upomega$  (59).

4 In cases of duplicated words owed to the expansion, we only kept their first occurrence.

5 https://dev.twitter.com/streaming/overview.

6 The streaming API receives a list of keywords and a language specification as input.

7 Available through http://www.translatum.gr.

8 The Python package gensim was employed (https://pypi.python.org/pypi/gensim).

9 Language recognition was performed using https://github.com/saffsd/langid.py.

10 https://translate.google.com.
